# Advances in Therapeutic Fc Engineering – Modulation of IgG-Associated Effector Functions and Serum Half-life

**DOI:** 10.3389/fimmu.2016.00580

**Published:** 2016-12-12

**Authors:** Abhishek Saxena, Donghui Wu

**Affiliations:** ^1^Laboratory of Antibody Engineering, Shanghai Institute for Advanced Immunochemical Studies, ShanghaiTech University, Shanghai, China

**Keywords:** antibody Fc region, ADCC, CDC, ADCP, serum half-life, aglycosylated antibody, FcRn, cancer therapy

## Abstract

Today, monoclonal immunoglobulin gamma (IgG) antibodies have become a major option in cancer therapy especially for the patients with advanced or metastatic cancers. Efficacy of monoclonal antibodies (mAbs) is achieved through both its antigen-binding fragment (Fab) and crystallizable fragment (Fc). Fab can specifically recognize tumor-associated antigen (TAA) and thus modulate TAA-linked downstream signaling pathways that may lead to the inhibition of tumor growth, induction of tumor apoptosis, and differentiation. The Fc region can further improve mAbs’ efficacy by mediating effector functions such as antibody-dependent cellular cytotoxicity, complement-dependent cytotoxicity, and antibody-dependent cell-mediated phagocytosis. Moreover, Fc is the region interacting with the neonatal Fc receptor in a pH-dependent manner that can slow down IgG’s degradation and extend its serum half-life. Loss of the antibody Fc region dramatically shortens its serum half-life and weakens its anticancer effects. Given the essential roles that the Fc region plays in the modulation of the efficacy of mAb in cancer treatment, Fc engineering has been extensively studied in the past years. This review focuses on the recent advances in therapeutic Fc engineering that modulates its related effector functions and serum half-life. We also discuss the progress made in aglycosylated mAb development that may substantially reduce the cost of manufacture but maintain similar efficacies as conventional glycosylated mAb. Finally, we highlight several Fc engineering-based mAbs under clinical trials.

## Introduction

**Figure 1:**
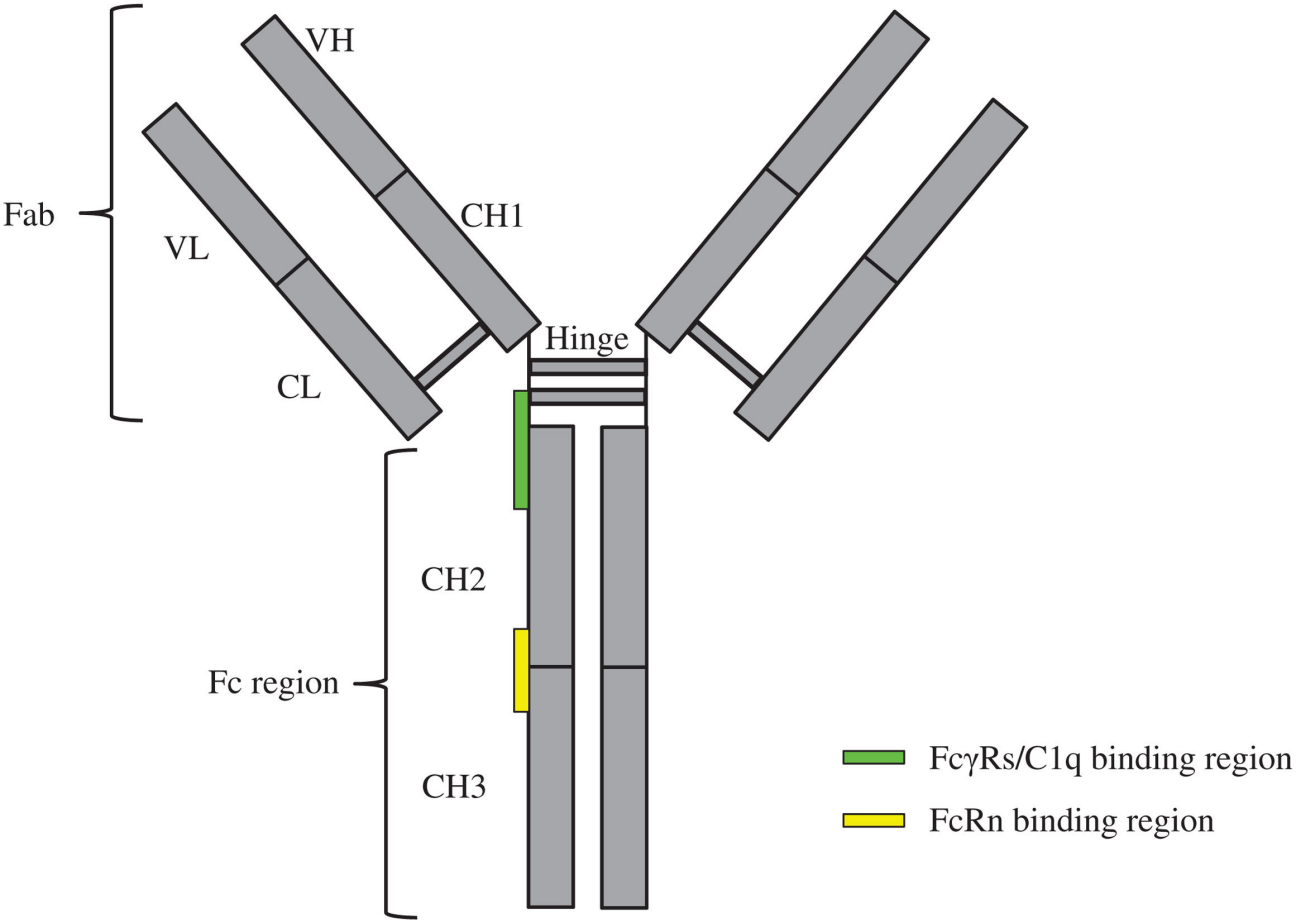
**Schematics of immunoglobulin gamma overall structure and its binding regions with FcγRs, C1q, and FcRn**. The constituent heavy [VH, CH1, hinge, CH2, and CH3 (gray)] and light chains [VL and CL (gray)] linked by inter-chain disulfide bonds are shown. The site at which FcγRs/C1q interacts with the crystallizable fragment (Fc) region is located in the lower hinge-upper CH2 (green rectangle); the site at which FcRn interacts with the Fc region is located in the interface of CH2–CH3 (yellow rectangle).

Monoclonal antibodies (mAbs) can target tumors through specific recognition of tumor-associated antigens and subsequent recruitment of effector elements including macrophages, dendritic cells, natural killer (NK) cells, T-cells, and the complement pathway components (1). Such recruitments are achieved by interactions among the immunoglobulin gamma (IgG)-crystallizable fragment (Fc) and the immune cell receptors like Fcγ receptors (FcγRs) and the complement protein C1q of the complement system (2–4). These interactions lead to the activation of immune cells for enhanced antibody-dependent cellular cytotoxicity (ADCC)/antibody-dependent cell-mediated phagocytosis (ADCP), formation of the membrane attack complex, and more efficient presentation of antigen to the dendritic cells (1). Through a recycling mechanism, the neonatal Fc receptor (FcRn) prolongs the half-life of mAbs in a pH-dependent interaction with the Fc region (5). The schematic of overall IgG structure and its binding regions with FcγRs, C1q, and FcRn is depicted in Figure 1.

The FcγRs, consisting of FcγRI (CD64), FcγRII (CD32), and FcγRIII (CD16) classes, are heterogeneous in terms of their cellular expression and Fc binding affinities (1, 6). FcγRI binds to the Fc region with *K*_D_ ~10^−8^–10^−9^ M and is expressed on mononuclear phagocytes, dendritic cells, and IFN-γ-activated neutrophils (1, 7, 8). FcγRII binds to the Fc region with relatively lower affinity (*K*_D_ ~10^−7^ M) and exists in five isoforms; among them, activating (FcγRIIa, harboring an immunoreceptor tyrosine-based activation motif on neutrophils) or inhibitory (FcγRIIb, harboring an immunoreceptor tyrosine-based inhibitory motif predominantly on B-lymphocytes) are critical for immune regulation (1, 7). FcγRIII, expressed in two isoforms, binds the Fc region with the lowest affinities (*K*_D_ ~10^−5^ M) (1, 7). Among these, FcγRIIIa has a moderate Fc binding allele (V158) and a low binding allele (F158), and is expressed on NK cells, macrophages, and T-cell subsets and activates NK and T cell-mediated ADCC response (1, 6, 7); FcγRIIIb is exclusively present on neutrophils and lacks signal generation capacity (1, 7).

**Figure 2:**
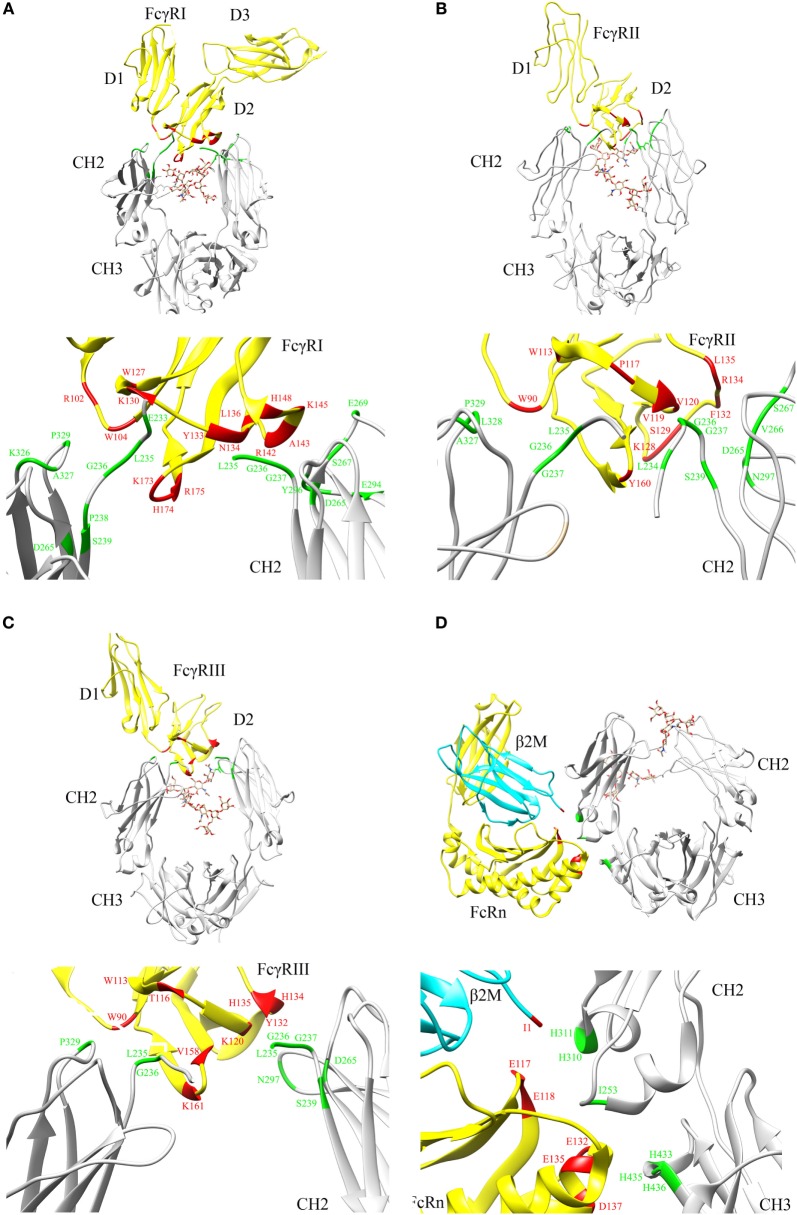
**Crystal structures illustrating crystallizable fragment (Fc) interactions with FcγRs and FcRn**. Representative structures are shown for **(A)** Fc–FcγRI cocrystals [PDB: 4W4O (9)], **(B)** Fc–FcγRII cocrystals [PDB: 3RY6 (10)], **(C)** Fc–FcγRIII cocrystals [PDB: 1T89 (11)], and **(D)** Fc–FcRn cocrystals [PDB: 1I1A (13)] with β2 microglobulin (β2M) domain shown in cyan. The Fc region and FcγRs are represented by gray and yellow color, respectively **(A–D)**. N297 glycans within the CH2 domain are shown in stick model. The critical binding regions are highlighted in the upper part of each panel; region from the Fc fragment in green, region from the FcγRs, FcRn, and β2M in red. The lower part of each panel shows the detailed residues, which are involved in the interactions between Fc and its binding partners.

Crystal structures of Fc in complex with FcγRI (9) (Figure 2A), Fc in complex with FcγRII (10) (Figure 2B), and Fc in complex with FcγRIII (11) (Figure 2C) reveal that the FcγRs’ interaction sites on Fc are all located within the lower hinge-upper heavy chain constant domain 2 (CH2). Furthermore, the binding affinity of Fc region to FcγRs also varies with the IgG subclasses (12).

The C1q is a multisubunit protein of the complement system (3). It uses one of its six heads to establish a low-affinity (~10^−6^ M) interaction with the lower hinge-upper CH2 domain of the Fc region (3). Crystal structure of the human C1q head revealed that it is assembled with a heterotrimer globular architecture (14). Though the molecular basis of how the C1q head recognizes the Fc region is not known at the atomic resolution, Schneider and Zacharias (15) proposed a working model of C1q in complex with Fc based on known experimental data, docking, and molecular dynamics simulation. According to this model, upon initial weak interaction between the C1q head and the Fc region, IgGs can aggregate while recognizing “multiple epitopes” on the antigen surface and thus give many C1q molecules an opportunity to bind to their Fc regions, which enhance the “cumulative affinity” to ~10^−9^ M (3, 15). This leads to the deposition of complement component 3 (C3b) on the target cell and ultimate formation of the membrane attack complex that disrupts the lipid bilayer of the target cell, promotes cytolysis, and completes complement dependent cytotoxicity (CDC) (16, 17).

The pharmacokinetic profiles of antibodies vary among subclasses and are related to the structural features of the Fc region (18, 19). It is known that the serum half-life of IgG subclasses (IgG1, IgG2, and IgG4) is ~23 days as compared to 2–6 days for IgG3 and other Ig classes (18, 19). The Fc region spanning the interface of CH2 and CH3 domains interacts with the FcRn in the placenta, liver, mammary glands, and adult intestine to regulate IgG homeostasis and deliver maternal IgG across the placenta to the fetus (5). This interaction is favored by an acidic environment of the endosome after IgG is pinocytosed and thus IgG is protected from lysosomal degradation (20). The endocytosed IgG is then recycled to the cell surface and released into the blood stream at an alkaline pH, thereby maintaining the sufficient IgG serum half-life for proper immune functions and desired therapeutic efficacies (20). Recently, the endothelial and hematopoietic cells are identified as the major sites associated with FcRn expression and their critical role in IgG homeostasis (20–23).

Based on site-directed mutagenesis of Fc region and design of a hybrid Fc heterodimer harboring one half of Fc wild type (WT) and one half of Fc mutant, Kim and coworkers identified the key Fc residues involved in the FcRn interaction and proposed a preliminary model that one Fc hinge homodimer bound with two FcRn molecules (24, 25). Shortly thereafter, Burmeister and coworkers reported high resolution crystal structure of FcRn alone at 2.2 Å and low resolution crystal structure of Fc in complex with FcRn at 6.5 Å (26, 27). Structural analysis and biophysical data confirmed that one Fc homodimer binds with two FcRn molecules (26–29). Later, Martin and coworkers reported a high resolution crystal structure of Fc in complex with FcRn at 2.8 Å (13). This structure clearly shows the key residues involved in Fc and FcRn interactions and reveals the pH-dependent binding mechanism (13) (Figure 2D).

Evidence demonstrates the presence of oligosaccharides, attached to the N297 residue within the CH2 domain Asn-X-Ser/Thr glycosylation motif of Fc region, is essential in maintaining the Fc conformation and mediating its interactions with FcγRs (FcγRI, FcγRIIa, FcγRIIb, and FcγRIIIa) and C1q, but not FcRn (30–41). The glycan moiety is formed by two N-linked biantennary oligosaccharide chains consisting of a core heptasaccharide [N-acetylglucosamine (GlcNAc) and mannose (Man)] but occurrence of other residues like terminal N-acetlyneuraminic acid, galactose (Gal), bisecting N-acetylglucosamine (GlcNAc), and fucose (Fuc) have also been reported (1, 42, 43). Additionally, 5–17 and 2–7% of IgG structures could be monosialylated and disialylated, respectively (1, 44). This imparts a significant complexity and heterogeneity to therapeutic IgG molecules when expressed in mammalian cells, which can affect the therapeutic profile of IgG (30). On the other hand, the presence of bisected N-acetylglucosamine structures in rituximab, a purer glycoform with lesser heterogeneity, leads to an efficient engagement of FcγRIII and increases ADCC activity against CD20^+^ cells by up to 20-fold (30, 45). Similarly, non-fucosylated glycoform of Herceptin produced in engineered Chinese hamster ovary (CHO) cell line (LEC13) can enhance ADCC *via* FcγRIII engagement by up to 50-fold (30, 46).

However, mAb-associated glycan heterogeneity poses several key challenges (30, 33, 45–51) including (1) difficulties in developing therapeutic mAbs with glycan composition similar to naturally occurring human IgG1, (2) difficulties in controlling glycan heterogeneity, (3) lengthier development time to construct cell lines producing glycan homogeneity, (4) lengthier IgG production time and higher manufacturing cost in mammalian cells as compared to that in *E. coli* or yeast-based expression systems, (5) dominance of particular glycoforms that can affect effector functions of IgG molecules, and (6) difficulties in separating various glycoforms generated from mammalian cells. Alternatively, development of aglycosylated mAbs with similar efficacy as glycosylated counterpart but lower manufacturing cost has attracted great efforts in the past decade.

In this review, we focus on the recent progress in therapeutic Fc engineering-associated effector functions (ADCC, ADCP, and CDC) and pharmacokinetics. The mutations known to induce profound effects on Fc interaction with FcγRs, C1q, and FcRn are summarized (see Table 1). We also briefly describe the advances in aglycosylated mAb development. Finally, we highlight clinical trials of several mAbs developed from relevant Fc engineering.

**Table 1:** **Tabulation of the Fc mutations known to mediate a profound effect on antibody effector functions and immunoglobulin gamma homeostasis**.

Fc type	Mutation	Target	Functional	Reference
Hu-IgG2-Glyco	K326W/E333S	C1q	Yes	(52)
Mu-IgG2b-Glyco	E235L	FcγRI	Yes	(2)
Hu-IgG3-Glyco	E235Y	FcγRI	Yes	(35)
Hu-IgG1-Glyco	S239D, I332E, S239D/I332E, and S239D/I332E/A330L	FcγRIIIa	Yes	(6, 53–55)
Hu-IgG1-Glyco	G236A, G236A/I332E, S239D/I332E, and G236A/S239D/I332E	FcγRIIa > FcγRIIIa > FcγRI	Yes	(56)
Hu-IgG1-Glyco	L235V/F243L/R292P/Y300L/P396L	FcγRIIa	Yes	(57, 58)
Hu-IgG1-Glyco	P238D/L328E	FcγRIIb	NA	(59)
Hu-IgG1/IgA-Glyco	IgGA (many motifs)	FcγRs + FcαRI	Yes	(60)
Hu-IgG1-Glyco	F243L/R292P/Y300L and F243L/R292P/Y300L/P396L	FcγRIIIa/FcγRIIa	Yes	(29)
Hu-IgG1-Aglyco	S298G/T299A	FcγRIIa	NA	(61)
Hu-IgG1-(-Fuc)	F234L	FcγRIIIa	Yes	(62)
Hu-IgG1-Aglyco	E382V/M428I	FcγRI	Yes	(63)
Hu-IgG1-Aglyco	Q295R/L328W/A330V/P331V/I332Y	FcγRI	Yes	(64)
Hu-IgG1-Glyco	M428L/N434S	FcRn	Yes	(65)
Hu-IgG1-Glyco	M252Y/S254T/T256E and H433K/N434F/Y436H	FcRn	Yes	(66, 67)
Hu-IgG1-Glyco	N343A/E380A	FcRn	Yes	(68)
Hu-IgG1-Glyco	M252Y/S254T/T256E	FcRn	Yes	(69)
Hu-IgG1-Glyco	T250R/M428L	FcRn	Yes	(70)
Hu-IgG1-Aglyco	Q295R/L328W/A330V/P331V/I332Y	FcRn	Yes	(64)

*Hu, Human; Mu, Murine; Glyco, Glycosylated; Aglyco, Aglycosylated; Fuc, Fucose; NA, not available*.

## Modulation of Effector Functions by Fc Engineering

To develop more effective antibodies with desired ADCC, ADCP, and CDC activities, various strategies including site-directed mutagenesis, alanine scanning, structure-based computational design, and directed evolution technologies are employed.

The Fc amino acid residues that confer improved binding to FcγRs/C1q and enhanced immune response were initially characterized by site-directed mutagenesis studies. The earliest described mutations were discovered by scanning residues to isolate non-binders while focusing on the conserved residues. Fc residues (E318, K320, and L322) in the mouse IgG2b-Fc region were identified as the C1q binding site (3). However, the relevance of E318 and K320 was challenged in human Fc–C1q interaction (71). Novel residues (D270, K322, P329, and P331) were proposed for normal C1q binding on human Fc (71). This finding underscores the interspecies differences in such molecular interactions that may show a different effect in preclinical models. Furthermore, an IgG1 isotype of rituximab carrying K326W/E333S mutations was shown to have fivefold more binding to C1q (52) and the same motif, when transferred to the IgG2 isotype (poor complement activator) of rituximab, increased the cell lysis by fivefold (52). Next, a single mutation from E to L at position 235 of the mouse IgG2b-Fc region proposed it to be the “major determinant” for FcγRI binding (with ~100-fold increased affinity to human monocyte FcγRI) (2). Additionally, using a mouse–human chimeric antibody, amino acids at position 234 and 237 were shown to mainly influence the interaction with FcγRII. Based on these observations, FcγRI and FcγRII were proposed to recognize an overlapping but non-identical site on the Fc region (35).

Alanine scanning mutagenesis of selected Fc residues resulted in many variants with altered binding to specific FcγRs, which was also reflected in their ability to promote ADCC. Activating FcγRIIIa mutations improved ADCC by 100% (68). Furthermore, mutants based on the activating or suppressing effect on FcγRs were categorized into different classes. Among these, IgG1 mutations A327Q/P329A (interact with FcγRI), D265A/S267A/H268A/D270A/K326A/S337A (interact with FcγRIIa), and T256A/K290A/S298A/E333A/K334A (interact with FcγRIIIa) promoted high-affinity interactions (68).

Computational optimization of the Fc region by creating a single (S239D or I332E), double (S239D/I332E), and triple mutations (S239D/I332E/A330L) improved the affinity against human FcγRIIIa^V158/F158^ allele by up to 169-fold (6). The mutations favoring Fc binding to activating (FcγRIIIa) receptor over the inhibitory (FcγRIIb) receptor are important to develop IgGs with better activating to inhibitory capacity (IIIa:IIb ratio), which was monitored using surface plasmon resonance. These mutations showed up to ninefold improvement in IIIa:IIb ratio and contributed to more than twofold enhancement in ADCC/ADCP activity, and the S239D/I332E double mutant significantly depleted CD20^+^ B cells *in vivo* compared to WT IgG (6). The same Fc mutations also enhanced *in vitro* ADCC/ADCP activity against lymphoma cell lines and directly translated into a more effective treatment of lymphoproliferative diseases when incorporated into anti-CD19/CD40 mAbs (53, 54). Furthermore, it was shown that a change from glycine to alanine at residue 236 can shift the immune balance toward activating FcγRIIa relative to inhibitory FcγRIIb (56). The coupling of G236A to either I332E or S239D/I332E had dual beneficial effect as these mutants not only improve FcγRIIa:FcγRIIb ratio but also enhance binding to FcγRIIIa by ~6- to 31-fold (56). These mutants had significantly improved NK cell-mediated ADCC and macrophage-mediated ADCP activity (56).

In addition, “shuffled variants” of anti-CD20/CD57 antibody were constructed by grafting the CH1/hinge and CH3 carboxyl-terminal of IgG1 into the Fc of IgG3 to retain both the ADCC activity from IgG1 and the CDC activity from IgG3 (72). It is known that IgG1 is the most potent ADCC activator, while IgG3 has highest potency to recruit complement system (72). Therefore, IgG1 and IgG3 Fc regions can complement one another to maximize the immune effector response. These variants with chimeric CH regions showed ~25–60% increase in ADCC and CDC activity compared to WT of IgG1 and IgG3 molecules (72). Furthermore, the CDC activity of humanized anti-CD20 IgG1 (ocrelizumab) was increased by ~23-fold while retaining normal IgG1 ADCC by combining a triple mutant (S267E/H268F/S324T) with earlier reported G236A/I332E in the CH2 domain (73).

Multiple mutations (L235V/F243L/R292P/Y300L/P396L) in the trastuzumab Fc region (MGAH22) increased the potency against low Her2-expressing cells *via* low-affinity FcγRIIIa^F158^ engagement (57). The same Fc motif was applied to the MGA271 mAb (anti-CD276), which targets B7-H3^+^ tumor cells and resulted in an increased binding to FcγRIIIa, enhanced ADCC, and potent antitumor activity in a renal cell carcinoma/bladder cancer xenograft mouse model (58). Recently, the immune activating potential of IgA *via* FcαRI engagement was exploited by developing IgG and IgA hybrid molecules “IgGA” through substituting α1 loop residues of CH_γ1_2/3 region with CH_α1_2/3 (60). The “IgGA” hybrid trastuzumab mediated an enhanced ADCC/ADCP activity against Her2 overexpressing cells and destroyed up to 50% SkBr3 breast cancer cells (*via* ADCC) and MDA-MB-453 cells (*via* ADCP) (60). Similarly, “IgGA” hybrid rituximab lysed ~70% of the CD20^+^ calcein-AM-loaded Raji tumor cells when compared to the WT counterparts (60).

A negative selection strategy was applied using yeast surface display to enrich Fc mutants exhibiting selective high affinity to FcγRIIIa (29). Among these isolates, F243L was predicted to make a direct contact with the carbohydrate portion, which can “influence sialylation and affect the quaternary structure” of the Fc domain (29). Additionally, R292P partially reduced the binding to FcγRIIa, while Y300L, in combination with other mutations (F243L/R292P/V305I/P396L), showed an ~10-fold less *K*_D_, 100-fold enhanced ADCC activity, and potency in a xenograft mouse model of ovarian and breast cancer (29). Furthermore, an Fc variant with three changes (F243L/R292P/Y300L) was also effective in increasing the rate of cytolysis by ~100-fold (29). In another report, human IgG1 Fc variants were generated by an error-prone PCR and ribosome display to select high-affinity aglycosylated binders to human FcγRIIIa using a solution phase method (62). The isolated Fc mutant (F243L) lacked Fuc residues in most oligosaccharide chains and exhibited an improved FcγRIIIa^V158/F158^ binding and enhanced ADCC as compared to WT Fc (62). Recently, an anti-EGFR antibody (S239D/I332E) was noted to elicit mononuclear cell-mediated ADCC *via* FcγRIIIa engagement and at the same time showed impaired polymorphonuclear cell (PMN)-mediated ADCC due to the engagement of FcγRIIIb, a highly homologous isoform to FcγRIIIa (55). The inability of FcγRIIIb to activate immune signaling in such a scenario can be overcome by imparting high-affinity binding to FcγRIIa, which can enhance both NK cell- and PMN mediated ADCC (55). These observations highlight that Fc engineering toward related FcγRs needs to be tailored specifically to achieve desirable immune effects.

## Modulation of Antibody Pharmacokinetics by Fc Engineering

Along with the efforts to engineer Fc regions for enhanced effector functions, attempts have been made to improve antibody pharmacokinetics. Clearly, enhanced Fc–FcRn interaction at acidic pH can extend IgG’s serum half-life and positively regulate its homeostasis, which may benefit patients by greater therapeutic efficacy, less frequent dosing, and lower cost burden. Alanine scanning and display/directed evolution are commonly used techniques to identify favorable Fc mutants that can strengthen Fc–FcRn pH-dependent interactions.

A human Fc variant (N434A), isolated by alanine scanning of all solvent-exposed residues, showed fourfold increased binding to FcRn at pH 6.0 (68), which later, when studied in cynomolgus monkeys, showed a twofold extension of IgG serum half-life confirming the modulation of pharmacokinetics (74). Such an interaction at pH 6.0 prolongs IgG availability in serum, which correlates with the therapeutic effectiveness as demonstrated by the improved antitumor activity of IgG-Fc mutant (M428L/N434S from the CH3 domain) in a human FcRn transgenic mouse model (65).

A phage displayed antibody library approach was employed with the aim of isolating high-affinity binders against FcRn at pH 6.0. Random mutations were created at residues T252, T254, and T256, which are proximal to the IgG–FcRn interaction site, and binders were selected in a solution phase against FcRn at pH 6.0 and eluted by PBS at pH 7.4 (66). The Fc mutants generated in this manner were able to bind both human and rat FcRn with high affinity at pH 6.0; however, these variants (M252Y/S254T/T256E from the CH2 domain and H433K/N434F/Y436H from the CH3 domain) also showed tighter binding to mouse FcRn at pH 7.4 that decreased the serum IgG concentration in a mouse model (67). Yeung and coworkers (74) reported a similar observation that an Fc mutant (N434W), though possessing an ~80-fold enhanced affinity to FcRn at both acidic and neutral pH, did not stabilize serum IgG due to the loss in pH selectivity. These results suggest that high-affinity binding to the receptor at neutral pH can compromise the “beneficiary effect” of increased affinity at pH 6.0. Later, a humanized anti-respiratory syncytial virus antibody (MEDI-524) with a triple mutant Fc (M252Y/S254T/T256E from the CH2 domain) was reported to have a 10-fold increase in a pH-dependent way toward FcRn and about 4-fold improvement in serum half-life in cynomolgus monkey (69). Similarly, a high-affinity Fc variant (T250R/M428L) bound FcRn selectively at pH 6.0 and accounted for a 2.8-fold lesser degradation of serum IgG2 (70) and IgG1 (75) in rhesus monkey. Accumulating studies highlight that to prolong IgG serum half-life, pH-dependent FcRn affinity has to be maintained.

Of note, Grevys and coworkers recently analyzed known IgG-Fc mutants, which show enhanced FcRn pH-dependent affinity and extended serum half-life (M252Y/S254T/T256E from the CH2 domain and M428L/N434S from the CH3 domain), for their effects on ADCC, ADCP, and CDC (65, 69, 76). Surprisingly, they found that both mutants showed reduced effector functions with regards to ADCC, ADCP, and CDC. More interestingly, they found one previously known mutant (H433K/N434F from the CH3 domain), which showed reduced FcRn pH-dependent affinity and shortened serum half-life, displayed enhanced effector functions in ADCP, CDC, and ADCC (76, 77). These findings highlight that, though the interaction region of Fc with FcγRs and C1q (lower hinge region-CH2 domain) is distant from its interaction with FcRn (CH2–CH3 domain interface), it is still possible that these mutants can trigger a long range effect to other part of the Fc region in an unknown mechanism.

## Aglycosylated Fc to Overcome Glycan Heterogeneity

The demand for therapeutic antibodies is high, and it has been estimated that 8,000 kg of clinical grade mAbs were produced in 2013 (78). Currently, ~50% of the clinical grade biologics are produced in mammalian cells like CHO, mouse myeloma cell lines NSO and SP2/0 (30, 78–80).

However, the inherent glycan heterogeneity of mAbs when expressed in mammalian cell systems can cause high production cost and variations of mAb functions from batch to batch. Recently, great efforts have been invested in developing aglycosylated mAbs as an alternative. Ideally, aglycosylated mAbs would be as efficient as or even better than its glycosylated peers in mediating effector functions for antigen or target cell clearance.

To develop aglycosylated IgGs as alternatives, Sazinsky and coworkers constructed three small subsets of saturation substitutions covering the Asn-X-Ser/Thr glycan motif of the Fc C′/E loop and displayed these libraries on the yeast cell surface (61). After selection against FcγRIIa by fluorescence-activated cell sorting (FACS), a double Fc mutant isolate (S298G/T299A) in an aglycosylated form showed threefold stronger binding as compared to the WT and variants with single mutation, which indicates that the glycosylation of N297 is not a strict requirement for the interaction of Fc with FcγRIIa (61). In contrast, the indispensable role of asparagine at position 297 was demonstrated in the backdrop of N297Q, N297D, or N297A mutation, which abolished the double mutant (S298G/T299A) binding to FcγRIIa (61). Moreover, this dual mutant is functional *in vivo* in that murine platelet clearance is as efficient as that of WT mAb. Based on the modeling of Fc dual mutants in complex with FcγRIIa, the N297 residue of an aglycosylated IgG can make a hydrogen bond with the S126 residue of FcγRIIa (61, 81). Furthermore, such interaction may be strengthened by a bridging water molecule present in an unbound FcγRII crystal (61, 81). However, this dual mutant showed 10-fold reduced FcγRI binding and no binding to both FcγRIII and C1q proteins. The inability of aglycosylated mAbs to bind with C1q and thus activate CDC can limit their application in treating hospital acquired microbial infections among cancer patients where complement activity plays an important role (82).

In another major breakthrough, bacterial display and FACS was used to isolate Fc variants displaying increased binding and specificity to FcγRI (63). One such aglycosylated variant called “Fc5” (E382V/M428I) was incorporated into trastuzumab. The trastuzumab-Fc5 variant bound selectively to the FcγRI with nanomolar range affinity and promoted monocyte-derived dendritic cell-dependent lysis of SkBr3 breast cancer cells that overexpress Her2. (63). The three-dimensional structure of human aglycosylated Fc domain suggests a greater “conformational flexibility of the CH2-CH3 domain interface,” as compared to the glycosylated counterpart (83). Additional mutations (Q295R/L328W/A330V/P331V/I332Y) in trastuzumab-Fc5 variant (63) increased the affinity for FcγRI by ~120-fold and retained pH-dependent FcRn binding and function (64). Other mutants were also reported with specific binding to FcγRI and without compromising the pH-dependent FcRn binding (84).

It is worth mentioning that removal of appended glycans, though does not affect IgG’s solubility, binding affinities to FcγRs, and *in vivo* half-life but often compromises IgG related CDC, lowers its thermostability and increases its aggregation at the low pH (85–87).

## Fc Engineering-Based mAbs Under Clinical Trials

A number of mAbs harboring various modifications in the Fc region are being investigated in different clinical trial stages (see Table 2 for Fc engineering-based mAbs being tested in clinics). These molecules broadly fall into three categories (1) with enhanced effector response to treat cancer and infectious diseases, (2) capable of inhibiting immune activation to treat inflammatory diseases, and (3) new class of aglycosylated mAbs with either inert or active-immune function.

**Table 2:** **Fc-engineered antibody candidates under clinical evaluation**.

Antibody	Target	Fc modification	Disease	Clinical development	Company	Reference
BI836826	CD37	NA	CLL	Phase-1	Boehringer	(6, 88, 89)
JNJ56022473	CD123	NA	AML	Phase-2	Janssen R & D	(89–91)
XmAb2513	CD30	NA	Hodgkin/large cell lymphoma	Phase-1	Xencor, Inc.	(89, 92, 93)
XmAb5871	CD19	S267E/L328F	SLE	Phase-1	Xencor, Inc.	(90, 94)
XmAb7195	IgE	S267E/L328F	Allergic diseases	Phase-1	Xencor, Inc.	(95)
XmAb5774	CD19	S239D/I332E	CLL	Phase-1	Xencor, Inc.	(89, 96)
TRX4	CD3	N297A	Type-1 diabetes mellitus (autoimmune)	Phase-3	GSK/Tolerx	(48, 49, 97)
Onartuzumab	MET	N297A	NSCLC/gastroesophageal cancer	Phase-3	Roche	(48, 98, 99)
ALD518	IL-6	N297A	RA/NSCLC/oral mucositis	Phase-2	Alder	(48, 49, 100)
TRX518	GITR	N297A	Malignant melanoma	Phase-1	Tolerx	(48, 49, 101)

*CLL, chronic lymphocytic leukemia; AML, acute myeloid leukemia; SLE, systemic lupus erythematosus; NSCLC, non-small cell lung cancer; RA, rheumatoid arthritis; NA, not available*.

*Source: https://clinicaltrials.gov/*.

The Fc variants capable of inducing enhanced ADCC are being tested in many antibody candidates. Anti-CD37 antibody (BI836826; Boehringer) against B cell malignancies is currently under phase-1 trial for the treatment of chronic lymphocytic leukemia (CLL) (6, 88, 89). This is a mouse–human chimeric antibody, which targets tetraspanin CD37 and shows high proapoptotic activity against malignant B cells *via* enhanced ADCC. Using human CD37 transgenic mice, a single dose of BI836826 was demonstrated to reduce peripheral B cells (88) and efficacious in suppressing tumor growth in Ramos mouse model of human B-cell lymphoma (88). Furthermore, therapeutic efficacy of a surrogate Fc-engineered antibody against macaque CD37 has also been demonstrated in cynomolgus monkey (88). A fully humanized anti-CD123 antibody (JNJ-56022473; Janssen R & D) targeting overexpressed interleukin-3 receptor α-chain is being tested in acute myeloid leukemia patients (89–91). The Fc fragment of JNJ-56022473 has been engineered for enhanced NK cell-mediated ADCC. The molecule efficiently reduced the growth of the patient-derived acute myelogenous leukemia xenografts in bone marrow and peripheral organs and increased the survival in animal models (91). Similarly, a humanized anti-CD30 antibody (XmAb2513; Xencor) with enhanced binding to FcγRIIIa is being evaluated in the treatment of CD30^+^ Hodgkin’s lymphoma (HL) patients who had previously received two or more therapies (89, 92, 93). It has been shown to be safely administered and biologically active in relapsed, refractory HL subjects and reduces tumor in a majority of patients (92, 93). Another interesting molecule is an anti-CD19 antibody (XmAb5774; Xencor), which induces potent NK cell-mediated ADCC/ADCP response against CLL (89, 96). The antibody is known to get internalized in primary CLL cells and induces a modest toxicity (96).

Antibodies harboring Fc mutations that can suppress the immune response are being tested for the treatment of inflammatory diseases. The immunosuppressive version of anti-CD19 antibody (XmAb5871; Xencor) binds inhibitory FcγRIIb with ~430-fold enhanced affinity and efficiently depletes CD19^+^ B-cells in systemic lupus erythematosus (SLE) patients (90, 94). The depletion of CD19^+^ B-cells correlates with the strong inhibition of B-cell receptor-induced calcium mobilization among healthy volunteers and SLE patients (94). The same Fc fragment has been engineered into a humanized anti-IgE antibody (XmAb7195; Xencor) for the treatment of allergies (95). This antibody prevents the binding of IgE to its high-affinity IgE receptor (FcϵRI) that is present on basophils and mast cells and is useful in the treatment of allergic asthma (95). The XmAb7195 has 5- and ~430-fold higher affinity for human IgE and FcγRIIb, respectively, and therefore is effective in inhibiting IgE production and plasma cell differentiation (95).

Finally, aglycosylated IgG molecules have recently been shown to have therapeutic properties (48, 49, 61, 63), and a few of them are undergoing clinical testing. An anti-CD3 antibody (TRX4; Tolerx) incorporating the N297A mutation suppresses pathogenic T-cells in type-1 diabetes (T1D) patients (48, 49, 97) and is being evaluated in phase-3 trials. The antibody downregulates pathogenic T-cells while restoring the normal activity of T-regulatory cells and thereby inhibits autoimmune mediated T1D (97).

It is widely accepted that hepatocyte growth factor (HGF) binding to receptor tyrosine kinase MET aggravates malignancy in a variety of cancers (98). Therefore, an aglycosylated anti-MET antibody (Onartuzumab; Roche) is being evaluated in phase-3 trials to inhibit the binding of HGF for treating lung and gastroesophageal cancers (49, 98, 99). This is an *E. coli*-derived humanized, affinity-matured antibody, which engages MET, thereby inhibiting HGF binding and receptor phosphorylation in HGF-dependent tumor models (98). Similarly, an aglycosylated mAb (ALD518; Alder) targeting IL-6 is being tested in phase-2 for a variety of diseases including rheumatoid arthritis, non-small cell lung cancer (NSCLC), and oral mucositis (48, 49, 100). The antibody was developed to inhibit proinflammatory cytokine IL-6 in oncogenic niches, which can otherwise lead to a cancer. The mAb ALD518 is reported to be well tolerated in phase-1 and -2 studies and ameliorated NSCLC-related anemia and cachexia (100). Another aglycosylated mAb (TRX518; Tolerx) is currently in phase-1 trials to treat malignant myeloma (48, 49, 101). TRX518 mAb recognizes the glucocorticoid-induced tumor necrosis factor receptor on regulatory and effector T-cells, B-cells, NK cells, and antigen-presenting cells to enhance effector T-cell response and inhibits T-regulatory cell-mediated suppression (101). Furthermore, the efficacy of TRX518 mAb in reducing tumor burden and increased survival rates has been demonstrated in mouse and non-human primate models (101).

These studies highlight that Fc engineering has played important roles in developing antibodies with desirable properties and functions, and the ongoing clinical studies can give valuable information on the efficacy of the Fc-engineered mAbs, as compared to their existing peers.

## Final Remarks

Crystallizable fragment engineering has made substantial progress in the identification of new mutant(s) that can enhance effector functions and improve pharmacokinetics of mAbs for cancer treatment. Two major notions have emerged during the past decades’ efforts. First, the ratio of human activating FcγRs (FcγRI, FcγRIIa, and FcγRIIIa) and inhibitory FcγR (FcγRIIb) has to be taken into account during Fc engineering design. Beneficial effect can be achieved when Fc mutant(s) show higher selectivity and binding affinity toward activating FcγRs, as compared to the inhibitory FcγR. Second, the synergistic effects of improved ADCC, ADCP, and CDC of Fc region could increase the potency in cancer treatment.

Excitingly, aglycosylated mAb, expressed in bacteria and yeast, have been found to possess similar properties as glycosylated mAb with regards to FcγRs binding and serum half-life. However, efforts are still needed to improve its thermostability, solubility, and its binding affinity to C1q and CDC activity before it can really compete with its glycosylated peer for cancer therapy. On the other hand, the inability of aglycosylated mAbs to bind to C1q may have beneficial effects when CDC activity is not required such as for treatment of autoimmune diseases where CDC is chronically and pathologically activated (102). Similarly, aglycosylated mAbs may have advantages over glycosylated counterparts when only selective activation of FcγRs is desired such as activation of FcγRI (63) or FcγRIIa (84) to stimulate tumor cell killing.

## Author Contributions

DW conceived the topic; AS and DW wrote the manuscript; and DW revised the manuscript.

## Conflict of Interest Statement

The authors declare that the research was conducted in the absence of any commercial or financial relationships that could be construed as a potential conflict of interest.
